# Cardiac Left Ventricular miRNA-26a Is Downregulated in Ovariectomized Mice, Upregulated upon 17-Beta Estradiol Replacement, and Inversely Correlated with Collagen Type 1 Gene Expression

**DOI:** 10.3390/ijms25105153

**Published:** 2024-05-09

**Authors:** Elishai Assayag, Irina Gurt, Einav Cohen-Kfir, Joshua Stokar, Donna R. Zwas, Rivka Dresner-Pollak

**Affiliations:** 1 Department of Endocrinology and Metabolism, Division of Medicine, Hadassah Medical Organization, Faculty of Medicine, Hebrew University of Jerusalem, Jerusalem 91120, Israel; elishai.assayag@gmail.com (E.A.); irina.gurt@mail.huji.ac.il (I.G.); einavck@ekmd.huji.ac.il (E.C.-K.);; 2 Linda Joy Pollin Cardiovascular Wellness Center for Women, Division of Cardiology, Hadassah Medical Organization, Faculty of Medicine, Hebrew University of Jerusalem, Jerusalem 91120, Israel; donnaz1818@gmail.com

**Keywords:** ovariectomy, C57BL/6 female mice, cardiac left ventricle, 17-β estradiol, miRNA-26a, *Col1α1*, *Col3α1*

## Abstract

Heart failure with preserved ejection fraction (HFpEF) is more prevalent in post- compared to pre-menopausal women. The underlying mechanisms are not fully understood. Data in humans is confounded by age and co-morbidities. We investigated the effects of ovariectomy and estrogen replacement on the left ventricular (LV) gene expression of pro-inflammatory and pro-fibrotic factors involved in HFpEF and putative regulating miRNAs. Nine-week-old C57BL/6 female mice were subjected to ovariectomy (OVX) or SHAM operation. OVX and SHAM groups were sacrificed 1-, 6-, and 12-weeks post-surgery (T1/SHAM; T1/OVX; T6/SHAM; T6/OVX, T12/SHAM). 17β-estradiol (E_2_) or vehicle (VEH) was then administered to the OVX groups for 6 weeks (T12/OVX/E2; T12/OVX/VEH). Another SHAM group was sacrificed 12-weeks post-surgery. RNA and miRNAs were extracted from the LV apex. An early 3-fold increase in the gene expression of *IL-1α*, *IL-6*, *Mmp9*, *Mmp12*, *Col1α1*, and *Col3α1* was observed one-week post-surgery in T1/OVX vs. T1/SHAM, but not at later time points. miRNA-26a was lower in T1/OVX vs. T1/SHAM and was inversely correlated with *Col1α1* and *Col3α1* expression 1-week post-surgery (r = −0.79 *p* < 0.001; r = −0.6 *p* = 0.007). miRNAs-26a, 29b, and 133a were significantly higher, while *Col1α1*, *Col3α1*, *IL-1α*, *IL-6*, *Tnfα*, *Mmp12*, and *FasL* gene expression was significantly lower in E_2_- compared to vehicle-treated OVX mice. miRNA-26a was inversely correlated with *Col3α1* in T12/OVX/ E_2_ (r = −0.56 *p* = 0.02). OVX triggered an early increase in the gene expression of pro-inflammatory and pro-fibrotic factors, highlighting the importance of the early phase post-cessation of ovarian function. E_2_ replacement therapy, even if it was not immediately initiated after OVX, reversed these unfavorable changes and upregulated cardiac miRNA-26a, previously unknown to be affected by menopausal status.

## 1. Introduction

Cardiovascular disease (CVD) is the leading cause of morbidity and mortality in men and women [1], but there are marked sex differences in its manifestations and outcomes. Whereas congestive heart failure overall is slightly more common in men, the incidence of heart failure with preserved ejection fraction (HFpEF) is twice as frequent in women when compared to men [2]. HFpEF is characterized by adverse cardiac remodeling, myocardial stiffness, fibrosis, and elevated left ventricular (LV) filling pressure. Co-morbidities, including hypertension, obesity, and diabetes, are associated with an increased risk of HFpEF [3], and each of these risk factors are associated with remodeling to a greater degree in women than in men. With aging, cardiac remodeling leads to increased LV wall thickness and decreased LV dimensions. However, women experience these changes at a more accelerated pace than men, starting in the peri-menopausal period [4,5].

The augmented risk of HFpEF in post-menopausal women is likely multi-factorial, with major contributions from aging and the withdrawal of estrogens. Deciphering the relative contributions of each of these factors is challenging due to their concurrent timing and inter-relationships [6]. Both aging and estrogens influence multiple pathways involved in HFpEF development, including extracellular matrix generation, inflammation, nitric oxide production, intra- and extra-cellular calcium homeostasis, the renin-angiotensin system, and mitochondrial function [7,8]. 

Ovariectomy (OVX) in rodents is an established animal model for studying human menopause [9]. However, most previous studies investigating cardiac changes induced by estrogen withdrawal in rodents have used additional insults beyond OVX to augment the cardiac response such as a high fat diet, pharmacologic or mechanical interventions to induce hypertension, genetic models of disrupted estrogen receptors, and advanced age [10,11,12,13]. These models do not capture the specific effects of estrogen deficiency per se.

Recent studies have demonstrated that the menopausal transition (MT) and the perimenopause, which begins with the onset of menstrual cycle irregularities or menopause-related symptoms and ends 12 months after cessation of the menstrual cycles, are associated with alterations in cardiometabolic and vascular health parameters linked to a significantly increased risk of developing cardiovascular disease [14].

The goal of this study was to investigate the time course of the transcriptional changes in gene coding for factors involved in cardiac remodeling and the putative microRNAs (miRNAs) regulating these factors in the heart left ventricle (LV), following estrogen withdrawal using the OVX mouse model. We tested the hypothesis that the resultant dramatic decrease in endogenous estrogens with ovariectomy without any additional insult is sufficient to induce an early adverse change in the LV gene expression of pro-fibrotic and pro-inflammatory factors. Moreover, we investigated the effects of estrogen replacement on the expression of these factors, even when estrogen was not immediately initiated after OVX. Interestingly, the most dramatic increase in gene expression of pro-fibrotic and pro-inflammatory factors occurred one week after OVX, highlighting the importance of this early phase, while estrogen replacement reversed the unfavorable expression signature even if started later after OVX. We revealed that LV miRNA-26a is influenced by estrogen status and is inversely correlated with LV *Col1α1* and *Col3α1* expression.

## 2. Results

### 2.1. OVX Triggers an Early Increase in the LV Gene Expression of Pro-Fibrotic and Pro-Inflammatory Factors

**Figure 1:**
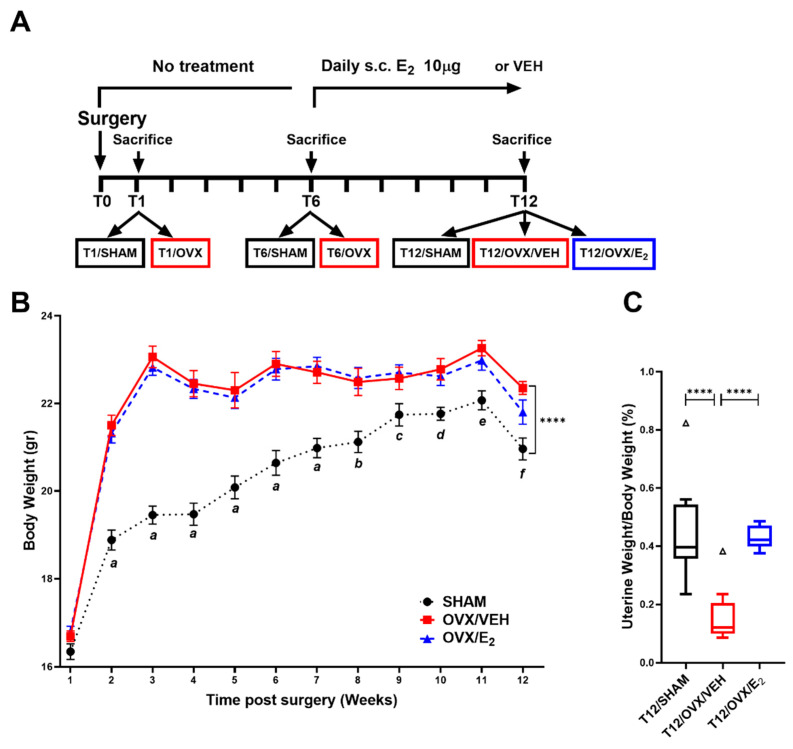
Study design, body, and uterine weight in SHAM, OVX, and E_2_-treated OVX mice. (**A**) Study design scheme (**B**) Body weight in T12/SHAM, T12/OVX/VEH, and T12/OVX/E_2_ mice over the course of 12 weeks (**C**) Uterine weight/body weight in T12/SHAM, T12/OVX/VEH, and T12/OVX/E_2_ mice. Boxes depict values from the 25th to 75th quartiles; the middle line depicts the mean, and the vertical whiskers show the 5th and 95th percentiles; the values outside this range are plotted as dots. Results are Mean ± SEM analyzed by repeated measures two-way ANOVA with Tukey’s post hoc correction; *a*, *p* < 0.0001 vs. T12/OVX/VEH and T12/OVX/E_2_; *b*, *p* < 0.001 vs. T12/OVX/VEH and *p* < 0.0001 vs. T12/OVX/E_2_; *c*, *p* < 0.05 vs. T12/OVX/VEH and *p* < 0.01 vs. T12/OVX/E_2_; *d*, *p* < 0.01 vs. T12/OVX/VEH and *p* < 0.05 vs. T12/OVX/E_2_; *e*, *p* < 0.001 vs. T12/OVX/VEH and *p* < 0.05 T12/OVX/E_2_; *f*, *p* < 0.0001 T12/OVX/VEH and *p* < 0.05 vs. T12/OVX/E_2_ (**B**) One-way ANOVA with Holm–Sidak’s post hoc correction (**C**). ****; *p* < 0.0001 vs. T12/SHAM (n = 10 mice/group). (SHAM = Sham operation; OVX= ovariectomy; VEH = Vehicle; E_2_ = 17-β estradiol). Triangles denote samples collected 12 weeks post OVX or SHAM operations.

The study design is depicted in Figure 1A. OVX mice had significantly higher body weights compared to SHAM mice throughout the course of the study (Figure 1B). E_2_ administration did not mitigate the weight gain in OVX mice (Figure 1B), possibly because it was initiated sometime after OVX and allowed weight gain to occur prior to treatment initiation. Uterine weight was significantly lower in OVX compared to SHAM mice, while E_2_ administration restored uterine weight to SHAM level (Figure 1C), confirming successful ovariectomy and efficacious estrogen replacement. 

**Figure 2:**
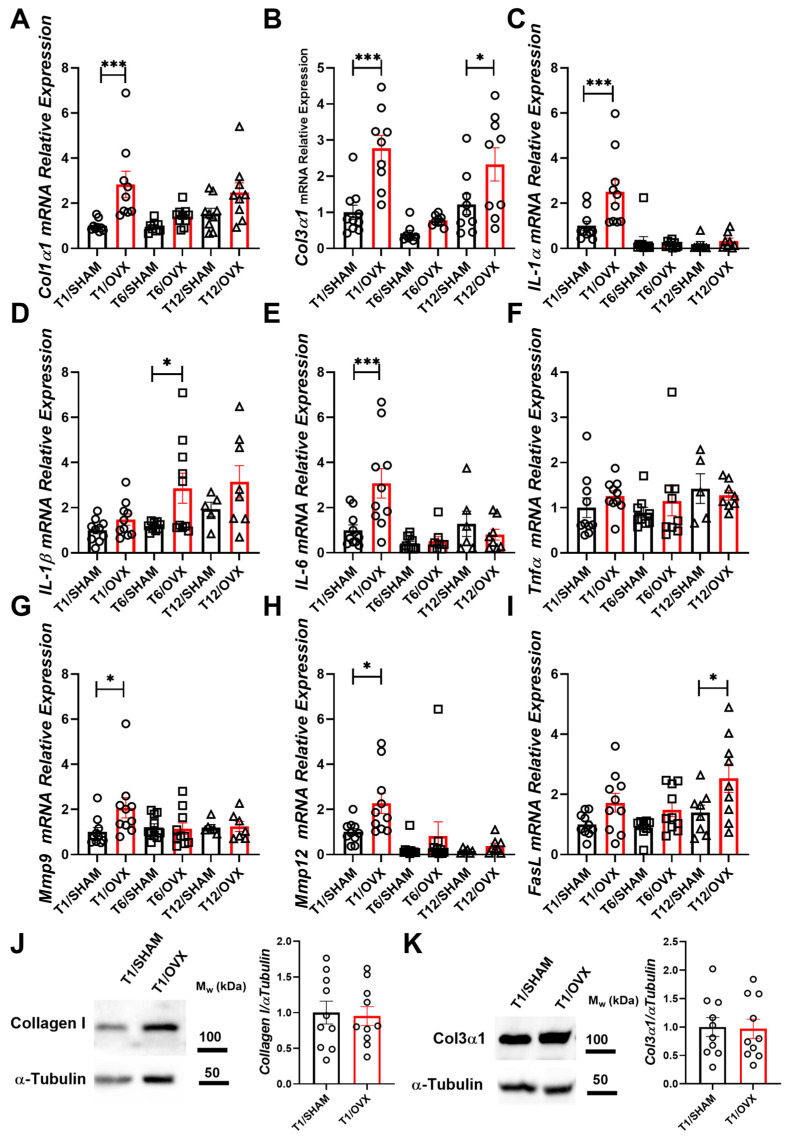
The effect of OVX on the expression of pro-fibrotic, pro-inflammatory, and apoptosis markers in the LV in OVX and SHAM mice 1-, 6-, and 12-weeks post-operation. (**A**–**I**) Relative Gene expression analysis of: (**A**) *Col1α1*; (**B**) *Col3α1*; (**C**) *IL-1α*; (**D**) *IL-1β*; (**E**) *IL-6*; (**F**) *Tnfα*; (**G**) *Mmp9*; (**H**) *Mmp12*; and (**I**) *FasL* in the LV normalized to the geometric mean of *Gapdh*, *actB*, and *Polr2a* expression (n = 6–10 mice/group) (**J**,**K**) Collagen I (**J**) and Col3a1 (**K**) protein level in LV extracts obtained from T1/OVX and T1/SHAM. Immunoblot of an image (left) and densitometry (right) (n = 10 mice/group). Results are Mean ± SEM analyzed by two-way ANOVA with Holm–Sidak’s post hoc correction (**A**–**I**) or by the Mann–Whitney test (**J**,**K**); * *p* < 0.05; *** *p* < 0.001 vs. SHAM mice. Circles denote samples collected 1 week, squares 6 weeks and triangles 12 weeks post OVX or SHAM operations.

OVX induced an early 3–4-fold increase in the LV gene expression of pro-fibrotic markers *Col1α1*, *Col3α1*, pro-inflammatory cytokines *IL-1α* and *IL-6*, and the metalloproteases *Mmp9* and *Mmp12* (Figure 2A–I). The effect of OVX on the expression of these markers was maximal one-week post-surgery and decreased over time. The expression of *FasL*, a marker of apoptosis, was significantly higher in OVX vs. SHAM mice 12-weeks post-operatively. No significant elevation in collagen 1 and col3a1 protein levels could be detected in OVX vs. SHAM mice (Figure 2J,K) using Western blots.

#### 2.1.1. OVX Induces an Early Decrease in LV miRNA-26a

Recent studies indicate that the cardiovascular cellular response is highly regulated via post-transcriptional mechanisms including non-coding short miRNAs [15]. miRNAs-133a, 29b, and 26a were shown to be negative regulators of cardiac fibrosis post-myocardial infarction and in cardiac hypertrophy [16,17,18,19] but were never investigated in the context of the gender-specific phenotypes of congestive heart failure. To understand the role of these miRNAs in the heart LV, in the context of OVX and estrogen replacement, we compared the expression of these miRNAs in OVX and SHAM mice.

**Figure 3:**
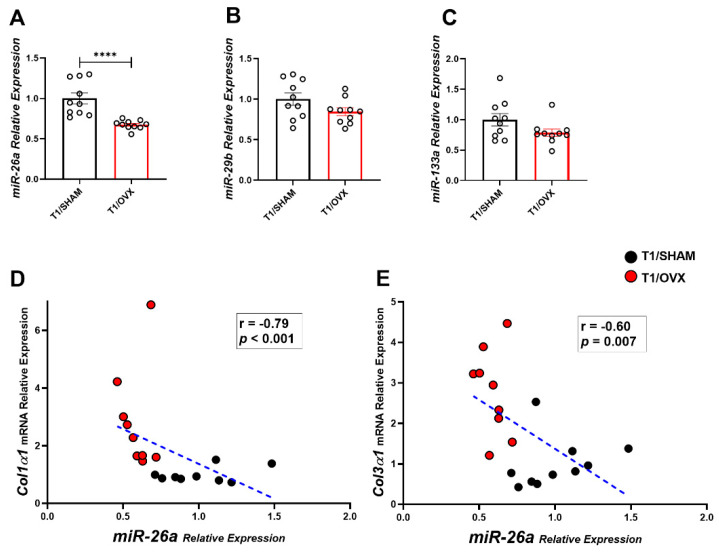
The effect of OVX on the expression of miRNAs-26a, 29b, and 133a in the LV in OVX and SHAM mice 1-week post-operation. (**A**–**C**) miRNA expression analysis of: (**A**) *miRNA-26a*, (**B**) *miRNA-29b*, and (**C**) *miRNA-133a* in T1/SHAM and T1/OVX mice normalized to the geometric mean of *U6* and *SnoRNA202* expression (n = 9–10 mice/group). (**D**,**E**) Scatter plots of the relative expression levels of: (**D**) *miRNA-26a* and *Col1α1 Col3α1* (**E**) in T1/OVX and T1/SHAM mice. Results are Mean ± SEM analyzed using the Mann–Whitney test (**A**–**C**), and Spearman correlation coefficient (**D**,**E**) **** *p* < 0.0001 vs. T1/SHAM mice. Circles denote samples collected 1 week post OVX.

Strikingly, a significant decrease in miRNA-26a was observed one week post-operatively in T1/OVX vs. T1/SHAM mice (Figure 3A). No difference was observed in miR-29b and miR-133a at this time point. (Figure 3B,C). Importantly, an inverse correlation between miRNA-26a and *Col3α1* and *Col1α1* relative gene expression was found at this time point (Figure 3D,E). Whether increased *Col3α1* expression is mediated in part via the reduced expression of anti-fibrotic miRNA-26a remains to be further investigated. There was no difference in the miRNA-26a level between OVX and SHAM mice at other time points. No significant difference in miRNAs-133a and 29b could be detected in OVX vs. SHAM mice at any time point.

#### 2.1.2. E_2_ Down-Regulates the LV Gene Expression of Pro-Fibrotic and Pro-Inflammatory Factors and Up-Regulates miRNAs-26a, 133a, and 29b in OVX Mice

**Figure 4:**
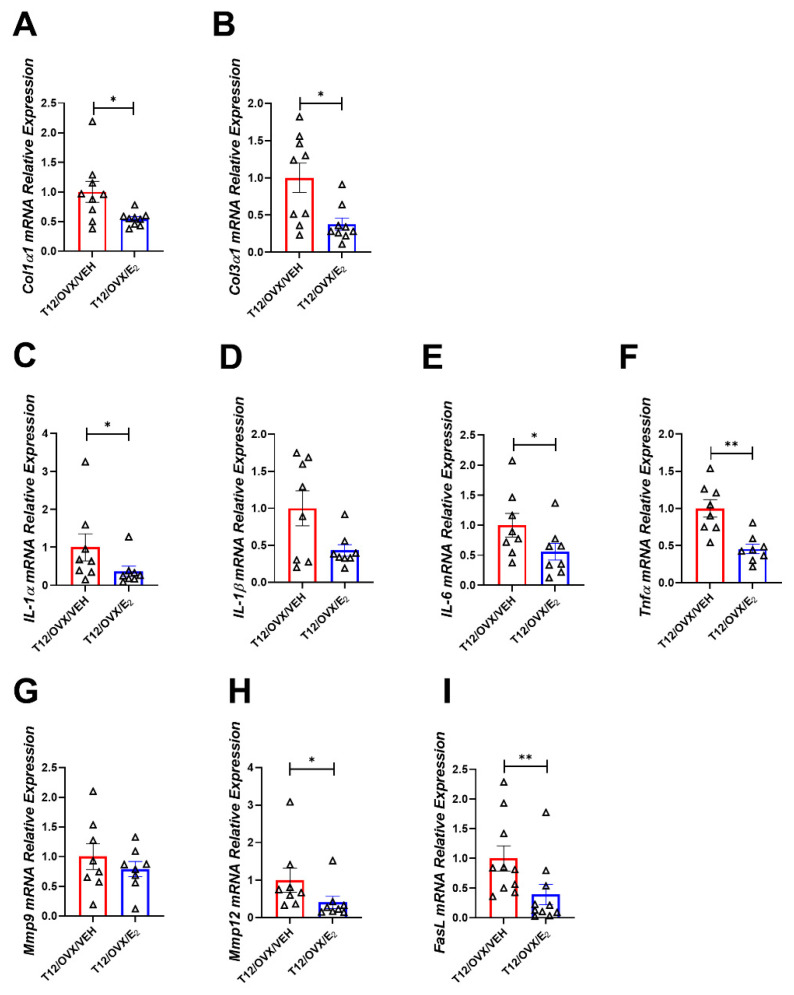
The effect of E_2_ administration on the LV gene expression of pro-fibrotic, pro-inflammatory, and apoptosis markers in OVX mice. Gene expression analysis of: (**A**) *Col1α1*; (**B**) *Col3α1*; (**C**) *IL-1α*; (**D**) *IL-1β*; (**E**) *IL-6*; (**F**) *Tnfα*; (**G**) *Mmp9*; (**H**) *Mmp12*; and (**I**) *FasL* in T12/OVX/VEH and T12/OVX/E_2_ normalized to the geometric mean of *Gapdh*, *actB*, *and Polr2a* expression (n = 8–10 mice/group). Results are Mean ± SEM analyzed using the Mann–Whitney test; * *p* < 0.05; ** *p* < 0.01 vs. T12/OVX/VEH. Triangles denote samples collected 12 weeks post OVX or SHAM operation.

**Figure 5:**
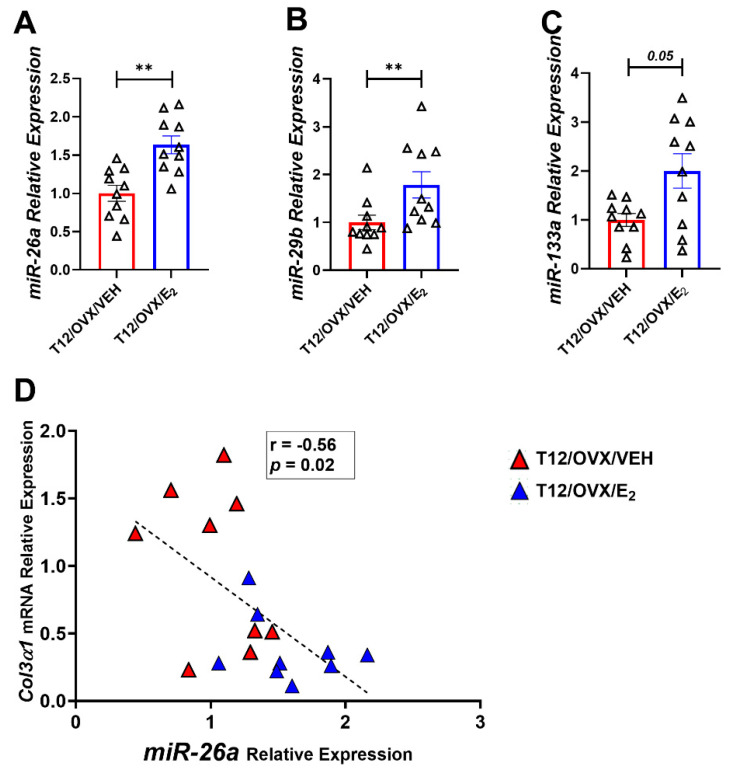
The effect of E_2_ administration on the expression of miRNA-26a, 29b, and 133a in the LV in OVX mice. (**A**–**C**) miRNA expression analysis of: (**A**) *miRNA-26a*; (**B**) *miRNA-29b*; and (**C**) *miRNA-133a* in T12/OVX/VEH and T12/OVX/E_2_ normalized to the geometric mean of *U6* and *SnoRNA202* (n = 10 mice/group). (**D**) Scatter plot of relative expression levels of *miRNA-26a* and *Col3α1* in T12/OVX/VEH and T12/OVX/E_2_ (n = 10 mice/group). Results are Mean ± SEM analyzed using the Mann–Whitney test (**A**–**C**), and Spearman correlation coefficient (**D**); ** *p* < 0.01 vs. T12/OVX/VEH. Triangles denote samples collected 12 weeks post OVX or SHAM operation.

E_2_ administration reversed the unfavorable effects of OVX on the gene expression of pro-fibrotic markers: *Col1α1*, *Col3α1*, the cytokines *Il-1α*, *Il-6*, *Tnfα*, the metalloprotease *Mmp12*, and the anti-apoptotic factor *FasL* (Figure 4A–I). Moreover, E_2_ administration up-regulated the expression of miRNAs-133a, 29b, and 26a (Figure 5A–C). Importantly, an inverse statistically significant correlation was observed between miRNA-26a and *Col3α1* expression (Figure 5D) in E_2_-treared OVX mice. Whether the beneficial effect of E_2_ on *Col3α1* is mediated in part via the up-regulation of the anti-fibrotic miR-26a remains to be investigated.

## 3. Discussion

This study demonstrates that OVX is sufficient in inducing an adverse LV gene expression signature with an increased expression of pro-fibrotic factors *Col1α1*, *Col3α1*, pro-inflammatory cytokines *IL-1α*, *IL-6*, and metalloproteases *Mmp9* and *Mmp12* in young adult female mice. The up-regulation of these genes occurred early, within one week of OVX. Moreover, *Col1α1* and *Col3α1* gene expression was inversely correlated with the level of miRNA-26a, previously reported to inhibit collagen expression in cardiomyocytes and fibroblasts under conditions of ischemic and hypertrophic cardiomyopathy but never reported to be affected by estrogen or a menopausal state [20]. Importantly, the administration of 17β-estradiol to OVX mice reversed these effects, reducing the gene expression of *Col1α1*, *Col3α1*, *IL-1α*, *IL-6*, *Tnfα*, *Mmp12*, and *FasL*, even though it was initiated six-weeks post-OVX. Finally, E_2_ administration up-regulated LV miRNA-133a, miRNA-29a, and miRNA-26a, previously shown to suppress adverse remodeling genes in myofibroblasts under the conditions of cardiac hypertrophy and myocardial infarction [16,21]. Our results demonstrate, for the first-time, cardiac changes in miRNA-26a, in the context of estrogen withdrawal and replacement.

The role of estrogens and their receptors in LV remodeling and fibrosis has been demonstrated in multiple studies in rats and mice [22]. In those studies, additional interventions beyond OVX were implemented to generate adverse cardiac remodeling and fibrosis, including transverse aortic constriction, a high-fat diet, pharmacologic interventions to induce hypertension, including the administration of angiotensin II, deoxycorticosterone acetate, and salt, as well as the genetic manipulations of estrogen receptors type alpha and beta [10,11,12,13]. To our knowledge, our study is the first to demonstrate that estrogen withdrawal alone is sufficient to induce an adverse LV gene and miRNA expression program.

A dramatic increase in pro-fibrotic and pro-inflammatory LV gene expression occurred as early as one-week post-surgery, and the differences between OVX and SHAM mice decreased over time. Our results highlight the accelerated pace of adverse changes in LV gene expression following the cessation of ovarian function. Importantly, our results show that, at least in young female mice, there is a favorable effect of E_2_ replacement on the LV gene expression of remodeling-related factors even if estrogen replacement is not immediately initiated after OVX. Moreover, our results suggest that OVX- and estrogen-induced changes in LV miRNA-26a may have a role in regulating LV collagen gene expression. To our knowledge, this is the first report of reduced LV miRNA-26a induced by OVX and the reversal upon E_2_ administration.

miRNAs are short non-coding RNAs that serve as post-transcriptional epigenetic regulators of gene expression. By binding to their target mRNA, miRNAs suppress mRNA translation into protein or promote its degradation. Estrogens were shown to regulate miRNA transcription and processing in multiple cells in the cardiovascular system (CVS). The binding of the estrogen–estrogen receptor complex to miRNA promoters and an enhancer upstream of the Dicer transcription start site up-regulates miRNA transcription and increases Dicer-induced pre-miRNA cleavage [14,22]. E_2_ was previously shown to reduce miRNAs-21, 27a,b 106a,b, and 23a in cardiomyocytes. These miRNAs were shown to increase the inhibitors of pro-fibrotic genes [23]. Our findings demonstrate an E_2_-mediated increase in miRNAs that directly reduces pro-fibrotic genes.

Sex differences in the cardiac expression of miRNAs were reported in healthy hearts and in cardiac fibrosis [21]. Reduced cardiac miRNAs-133a and 29b was previously reported in OVX rats [24,25] However, their upregulation by E_2_ replacement has never been reported so far. Moreover, an OVX-induced decrease in cardiac miRNA-26a has never been shown nor its up-regulation by estrogen administration.

The role of the miRNA-26 family in the CVS is complex. It was shown to affect multiple cells including vascular endothelial cells, smooth muscle cells, cardiac fibroblasts, and cardiomyocytes [16]. In endothelial cells, miRNA-26a was reported to regulate physiologic and pathologic angiogenesis by modulating BMP/SMAD1 signaling [16]. Furthermore, miRNA-26a suppressed collagen 1 and CTGF gene expression in cardiac fibroblasts in vitro and in vivo in mice subjected to aortic constriction. miRNA-26a was suggested to be regulated by NF-κB [20]. Indeed, miRNA-26a–deficient mice have evidence of myocardial fibrosis, whereas the overexpression of miRNA-26a inhibited myocardial fibrosis in vivo and angiotensin II-induced fibrogenesis in cardiac fibroblasts [26].

The miRNA-29 family was reported to have a protective role against cardiac fibrosis by repressing genes coding for extra-cellular matrix proteins including collagens, fibrillins, and elastin [18]. The down-regulation of miRNA-29 was reported in the cardiac region adjacent to the infarct zone after myocardial infarction [18].

miRNA-133a is highly abundant in cardiomyocytes and affects several molecular pathways involved in cardiac remodeling [17,27]. It decreases collagen1α1 expression [28] and directly down-regulates CTGF [28]. The inhibition of miRNA-133a leads to cardiac hypertrophy [29], while its over-expression attenuates the hypertrophic response to phenylephrine [30]. In an animal model of diabetic cardiomyopathy-induced fibrosis, there was a dramatic decrease in miRNA-133a expression, accompanied by increased TGF-β [15]. Intriguingly, miRNA-133a was suggested as an inducer of cardiac regeneration via reprogramming cardiac fibroblasts into cardiomyocyte-like cells [31]. Importantly, in patients undergoing coronary bypass grafting, the decreased expression of miRNA-133 in the right atrium was associated with heart failure severity as determined by the NT-proBNP level [32]. Conversely, patients with higher myocardial miRNA-133a expression were more likely to normalize LV mass after aortic valve replacement [33]. Finally, lower levels of miRNA-133a were found in peripheral blood mononuclear cells in patients with LV diastolic dysfunction compared to the normal controls [34].

This study is not without limitations. The abrupt nature of surgical menopause differs from the more gradual process of physiologic menopause. Similarly, estrogen replacement was administered to young mice, which may lead to different effects than what would be seen with estrogen replacement in older myocardium. To detect changes in collagen type 1 and 3 proteins, which are present primarily in the extracellular matrix immunohistochemistry, immunofluorescence or Masson Trichome staining may have been more appropriate. Unfortunately, we had no more tissue left to perform this staining, as we insisted on studying the LV apex. Finally, the physiologic significance of our gene expression analyses cannot be fully interpreted as cardiac structural parameters, such as heart weight or echocardiographic evaluation, were not performed. Future studies in pre-, peri-, and post-menopausal women are warranted to investigate the role of miRNAs-26a, 29b, and 133a as biomarkers and potential therapeutic targets in OVX- and menopause-associated cardiac changes.

## 4. Materials and Methods

### 4.1. Animals and Study Design

In total, 70 8-week-old C57BL/6J female mice were purchased from Harlan (Rehovot, Israel), housed in constant temperature rooms with 12 h light/dark cycle, and were allowed free access to Harlan Teklad chow and water. The 9-week-old mice were randomly subdivided into 7 groups (n = 10 mice/group): 4 groups were subjected to bilateral ovariectomy (OVX) and 3 groups were subjected to sham operations (SHAM). A group of each OVX and SHAM animals were sacrificed 1- and 6-weeks post-surgery (T1/OVX, T1/SHAM, T6/OVX, T6/SHAM, respectively; Figure 1A, study design scheme). Of the remaining 2 groups of OVX mice, one group was randomly assigned to receive daily subcutaneously (s.c.) 17-β-estradiol (E_2_) (10 µg) and the other OVX group received a vehicle for 6 weeks. E_2_- and vehicle-treated OVX and one additional SHAM group were sacrificed 12-weeks post the initial surgical procedure (T12/OVX/VEH, T12/OVX/E_2_, T12/SHAM, respectively, Figure 1A). Body weight was determined weekly. Mice were fasted for 12 h and killed by CO_2_ inhalation. Upon sacrifice, uterine weight was determined to confirm a successful ovariectomy. RNA and protein were extracted from the left ventricle (LV) apex and kept in −80 °C until analyzed. All animal experiments complied with the ARRIVE guidelines. The study was approved by The National Council for Animal Experimentation (approval number IL-10-12-113).

### 4.2. Gene Expression Analysis

RNA was extracted using BIO-TRI RNA (cat. No. 0090010233100, Bio-Lab, Lawrenceville, GA, USA), reverse transcribed into cDNA using the qScript cDNA Synthesis Kit (cat. 95047-100, Quanta Biosciences, Gaithersburg, MD, USA), and analyzed with SYBR Green-based quantitative Real-Time PCR in triplicates. Relative mRNA expression was determined by the comparative CT method and was normalized to the geometric mean expression of *Gapdh*, *actB* and *Polr2a*. For comparison of gene expression in SHAM versus OVX mice at 1-, 6-, and 12-weeks post-surgery, gene expression was further normalized to its expression in SHAM mice 1 week post-operation.

### 4.3. microRNA Analysis

miRNA-specific cDNA was generated with TaqMan^TM^ microRNA Reverse Transcription Kit (4366596, Applied Biosystem, Cartsbad, CA, USA). Relative miRNA expression was determined using the comparative CT method and normalized to the geometric mean expression of *U6* (TaqMan^TM^ microRNA assay: ID 001973) and *SnoRNA202* (TaqMan^TM^ microRNA assay: ID 001232) by using miRNA-specific primers: *miRNA-29b*-3p (mmu481300_mir), *miRNA-26a*-5p (mmu481013_mir), *miRNA-133a*-5p (mmu481498_mir).

### 4.4. Protein Analysis

Antibodies to the following proteins were used for Western blotting: Collagen I (ab34710, Abcam, Cambridge, UK), Col3a1 (sc-271249, Santa Cruz, Santa Cruz, CA, USA). Alpha Tubulin (ab89984, Abcam, UK) was used as loading control.

### 4.5. Statistical Analysis

Statistical analysis was performed using GraphPad Prism 6.01. Data analysis was performed with repeated measures two-way ANOVA, followed by Tukey’s post hoc correction to compare body weight in T12/SHAM, T12/OVX/VEH, and T12/OVX/E_2_ mice over the course of the study. One-way ANOVA, followed by Holm–Sidak’s post hoc correction, was used for uterine weight comparisons. Two-way ANOVA was used for the comparison of multiple groups means in OVX and SHAM mice. The Mann–Whitney test was used to assess differences between two groups that did not have a normal distribution. Correlation was assessed using the Pearson correlation coefficient analysis. Data is presented as Mean ± SEM. * *p* < 0.05 was defined as significant.

## 5. Conclusions

Our findings in ovariectomized young adult female mice suggest that estrogen deficiency per se promotes an unfavorable remodeling gene and miRNA expression program in the left ventricle that rapidly occurs after estrogen withdrawal and is partially ameliorated upon E_2_ administration. E_2_ administration increases the expression of the fibrosis-suppressing miRNAs 26a, 133a, and 29b. Future studies of the association between miRNAs-26a, 133a, and 29b and cardiac structural and functional parameters may increase our understanding of the mechanisms underlying changes in heart function in the transition from pre- to post-menopause. The role of these miRNAs as biomarkers and therapeutic targets remains to be explored.

## Data Availability

Data will be available upon request.
